# Demographic and regional trends of acute myocardial infarction-related mortality among young adults in the US, 1999–2020

**DOI:** 10.1038/s44325-025-00046-w

**Published:** 2025-03-04

**Authors:** Haibin Li, Jin Zheng, Frank Qian, Xinye Zou, Siyu Zou, Zhiyuan Wu, Xiuhua Guo, Pixiong Su

**Affiliations:** 1 Department of Cardiac Surgery, Beijing Chaoyang Hospital, Capital Medical University, Beijing, China; 2 Heart Center & Beijing Key Laboratory of Hypertension, Beijing Chaoyang Hospital, Capital Medical University, Beijing, China; 3 Beijing Municipal Key Laboratory of Clinical Epidemiology, Capital Medical University, Beijing, China; 4 Department of Radiology, University of Cambridge, Cambridge, CB2 0QQ UK; 5 Section of Cardiovascular Medicine, Boston Medical Center and Boston University Chobanian & Avedisian School of Medicine, Boston, MA USA; 6 Cambridge Institute of Public Health, University of Cambridge, Cambridge, CB2 8PQ UK; 7 Department of Epidemiology, Johns Hopkins Bloomberg School of Public Health, 615N. Wolfe Street, Baltimore, MD 21205 USA; 8 Department of Nutrition, Harvard T.H. Chan School of Public Health, Boston, MA USA; 9 Department of Epidemiology and Health Statistics, School of Public Health, Capital Medical University, Beijing, China

**Keywords:** Cardiology, Cardiovascular diseases

## Abstract

Little is known about how acute myocardial infarction (AMI) mortality has changed over the past two decades in young US adults. We aimed to evaluate the AMI mortality trend among young adults in the US from 1999 to 2020. Data were extracted from the US Centers for Disease Control and Prevention Wide-Ranging Online Data for Epidemiologic Research (CDC WONDER) database. Young adults aged 15–44 years with AMI listed as a contributing or underlying cause of death were included. Joinpoint regression was used to estimate the annual percent changes (APCs) and average annual percentage changes (AAPCs) in AMI-related age-adjusted mortality rates (AAMRs). There were 81,272 AMI-related deaths among young adults. The overall AAMR shows a stable trend from 1999 to 2003 (APC 0.2%); a steeper decrease from 2003 to 2009 (APC -4.2%); a steady decrease from 2009 to 2018 (APC -1.8%); and a significant increase from 2018 to 2020 (APC 7.3%); with an AAPC of -1.3% per year (95% CI -2.0% to -0.5%). The AAMR significantly decreased among men (AAPC -1.5%; 95% CI -2.2% to -0.7%), women (AAPC -1.0%; 95% CI -2.4% to -0.4%), non-Hispanic White (AAPC -1.9%; 95% CI -2.6% to -1.1%), and non-Hispanic or African American (AAPC -1.2%; 95% CI -1.8% to -0.6%). Across all geographical regions, the AAMR declined, most significantly in large metropolitan areas (AAPC -1.8%; 95% CI -2.7% to -0.8%). In conclusion, AMI mortality rates among young adults mostly declined from 1999 to 2020, with significant demographic and geographic variation.

## Introduction

Deaths from cardiovascular diseases (CVDs) increased from about 17.8 million in 2017 to 19.1 million in 2020 worldwide, with ischemic heart disease (IHD) remaining the leading cause and accounting for approximately half of total deaths from CVD^1,2^. In the past several decades, advances in the diagnoses and treatments of acute myocardial infarction (AMI) have led to a sharp decrease in mortality from IHD in high-income countries^3,4^. However, recent data suggest that the trends in AMI mortality rate among young adults are not declining in the same fashion as seen for middle-aged and older adults in the United States^5,6^. This is particularly concerning because AMI in young adults often leads to significant premature morbidity and mortality, creating substantial societal and economic burdens. The disparity also highlights the underlying differences in risk factors between young and older populations, as AMI in young adults tends to involve the rupture of premature atherosclerotic plaque and drug abuse^7,8^. There also exists significant disparities in mortality rates from AMI by sex, race/ethnicity, geographic region, and urban/rural status, likely due to differences in cardiovascular risk factors and incidence/prevalence of CVD^2,5,6^. Previous studies have shown inequality and an increased risk of premature mortality in populations with low socioeconomic status^9^. The indirect costs from lifetime productivity loss and economic dependency due to premature morbidity can further exacerbate these inequalities. Moreover, the outbreak of the COVID-19 pandemic in 2020 has profoundly affected the global healthcare system with a significant drop in cardiovascular preventative services as well as the direct impact of COVID-19 infection on cardiovascular morbidity/mortality^10^.

With advancements in the diagnosis and treatment of AMI, the burden of AMI in the young population might be underestimated due to potential differences in risk factor profiles and healthcare access patterns. Early identification and intervention in this demographic could prevent long-term health complications and reduce the cumulative socioeconomic burden and inequalities. Accordingly, this study focuses on young adults (<45 years of age) to examine the trends in AMI mortality over 20 years and better understand these patterns and their broader implications for public health.

## Methods

We extracted deaths occurring within the United States related to AMI from the US Centers for Disease Control and Prevention Wide-Ranging Online Data for Epidemiologic Research (CDC WONDER) database^11^. The Multiple Cause-of-Death Public Use record death certificates were used to select AMI-related deaths, which were identified as those with AMI reported anywhere on the death certification either as a contributing or underlying cause of death. AMI-related mortality in young adults was determined using the *International Statistical Classification of Diseases and Related Health Problems-10th Revision (ICD-10) codes I21-I22*^12,13^. According to a previous study^14^, we defined young adults as those who were aged 15–44 years at the time of death. This study did not require institutional review board approval given the deidentified nature of the database.

### Data abstraction

We extracted data on age, sex, race/ethnicity, population size, year, location of death, region, state, and urban-rural classification from the CDC WONDER database. Race/ethnicity was defined as Hispanic or Latino, non-Hispanic White, non-Hispanic Black or African American, non-Hispanic American Indian or Alaska Native, and non-Hispanic Asian or Pacific Islander. The location of death was classified as medical facilities (outpatient, emergency room, inpatient, death on arrival, or status unknown), home, hospice, and nursing home/long-term care facility. Regions were classified into Northeast, Midwest, South, and West. Based on the National Center for Health Statistics Urban-Rural Classification Scheme, counties were divided into rural and urban (large metropolitan area, medium/small metropolitan area) per the 2013 US census classification^15^.

### Statistical analysis

Crude and age-adjusted mortality rates (AAMRs) per 100,000 persons were first calculated by year, sex, race/ethnicity, states, regions, and urban-rural status with 95% CI, as previously described^12–14^. AAMRs were calculated by standardizing the AMI-related deaths to the 2000 US population^16^. The Joinpoint Regression Program (Joinpoint V 5.3.0 available from the National Cancer Institute) was used to determine trends in AMI mortality among young adults from 1990 to 2020^17^. This software uses Monte Carlo Permutation testing to describe data using the least number of segments possible while maintaining significance. This method detects significant changes in the trends by fitting log-linear regression models, where potential joinpoints are determined by using a grid search method. The program starts with a defined minimum number of joinpoints (0 in our study) and tests up to a defined maximum number of joinpoints (4 in our study). Annual percentage changes (APCs) with 95% CIs were calculated using the Monte Carlo permutation test among intervals identified by the Joinpoint regression. The weighted averages of the APCs were reported as average APCs (AAPCs) and 95% CIs as a summary of the reported mortality trend across the study period. To compare AAPCs among the subgroups during the study period, we set the subgroup with the lowest AAPCs as reference and analyzed the difference between each subgroup to the referent subgroup by permutation test and pairwise comparison. We considered that APC was increasing or decreasing if the slope describing the change in AAMR over time was significantly different than 0 using 2-tailed *t*-testing. *P* < 0.05 was considered statistically significant. To compare state-level AAMR across the study period, we used a Z-test to compare the difference between each state and the state with the highest AAMR, which served as the reference group, given by the equation $z=\,\frac{{{rate}}_{1}-{{rate}}_{2}}{\sqrt{{{SE}}_{1}^{2}+{{SE}}_{2}^{2}}}$, where$\,{{rate}}_{1}$ and ${{rate}}_{2}$ are the two estimates and ${{SE}}_{1}^{2}$ and ${{SE}}_{2}^{2}$ the corresponding standard errors. Sensitivity analyses were performed to include only adults aged 25–44 years, as well as using AMI only as an underlying cause of death. Data were analyzed from January 2023 to December 2024.

## Results

Between 1999 and 2020, a total of 3,522,871 all-cause deaths occurred in young adults, with 81,272 (2.3%) attributed to AMI (Supplementary Table 1). Of these, 23,474 (28.9%) were among women and 57,798 (71.1%) were among men. Additionally, 54,379 (66.9%) were non-Hispanic White, 17,022 (20.9%) were non-Hispanic Black, 980 (1.2%) were non-Hispanic American Indian or Alaska Native, 1,835 (22.6%) were non-Hispanic American Indian or Alaska Native, and 6,859 (8.4%) were Hispanic. Information for the location of death was available for 76,392 deaths. Of these, 53,220 (69.7%) occurred within medical facilities, 1,215 (1.6%) occurred in nursing homes/long-term care facilities, 248 (0.3%) occurred in hospices, and 21,709 (28.4%) occurred at home (Supplementary Table 2). The overall AAMR during the study duration (1999–2020) was 3.15 (95% CI 3.12–3.17).

### Annual trends for AMI-related mortality

**Fig. 1:**
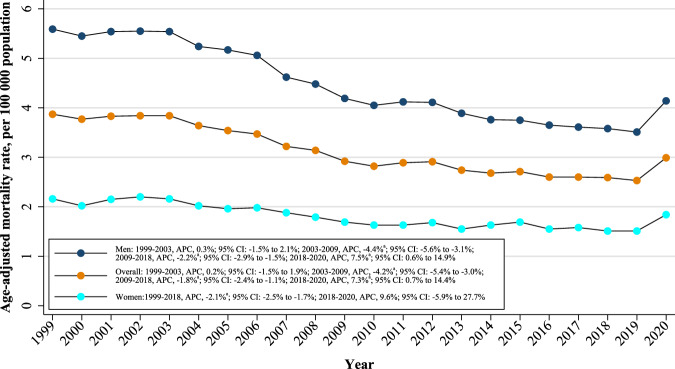
Overall and sex-stratified trends of age-adjusted and acute myocardial infarction-related mortality rates among adults aged 15–44 years from 1999 to 2020 in the United States. ^a^Indicates that the annual percentage change (APC) is significantly different from 0 at α = 0.05.

Overall, the AAMR for AMI-related deaths in young adults was 3.87 (95% CI: 3.76–3.98) in 1999 and 2.99 (95% CI: 2.89–3.09) in 2020. The overall AAMR shows a stable trend from 1999 to 2003 (APC: 0.2%; 95% CI: -1.5% to 1.9%); a steeper decrease from 2003 to 2009 (APC: -4.2%; 95% CI: -5.4% to -3.0%); a steady decrease from 2009 to 2018 (APC: -1.8%; 95% CI: -2.4% to -1.1%); and finally, a significant increase from 2018 to 2020 (APC: 7.3%; 95% CI: 0.7% to 14.4%) (Fig. 1 and Table 1). Between 1999 and 2020, the AAPC in age-adjusted AMI mortality rate among young adults was -1.3% per year (95% CI: -2.0% to -0.5%) (Table 1). A similar trend was observed in sensitivity analysis for adults aged 25–44 years (Supplementary Table 3 and Supplementary Fig. 1). No statistically significant difference in crude mortality trends was observed between the 15–25 and 25–44 year age groups (*P* = 0.456) (Supplementary Fig. 2). Using AMI as an underlying cause of death, a similar trend was seen in Supplementary Tables 4 and Supplementary Fig. 3.

**Table 1:** APCs and AAPCs in acute myocardial infarction-related mortality rates among adults aged 15–44 years from 1999 to 2020 in the United State

	Period 1		Period 2		Period 3		Period 4		AAPC for whole period (95% CI), %	Pairwise comparison for subgroup, *P*-value
	Years	APC (95% CI), %	Years	APC (95% CI), %	Years	APC (95% CI), %	Years	APC (95% CI), %		
Overall	1999–2003	0.2 (−1.5, 1.9)	2003–2009	−4.2 (−5.4, −3.0)^a^	2009–2018	−1.8 (−2.4, −1.1)^a^	2018–2020	7.3 (0.7, 14.4)^a^	−1.3 (−2.0, −0.5)^a^	NA
Sex										
Men	1999–2003	0.3 (−1.5, 2.1)	2003–2009	−4.4 (−5.6, −3.1)^a^	2009–2018	−2.2 (−2.9, −1.5)^a^	2018–2020	7.5 (0.6, 14.9)^a^	−1.5 (−2.2, −0.7)^a^	Reference
Women	1999–2018	−2.1 (−2.5, −1.7)^a^	2018–2020	9.6 (−5.9, 27.7)	NA	NA	NA	NA	−1.0 (−2.4, −0.4)^a^	0.001
Race/Ethnicity										
NH White	1999–2002	1.0 (−4.4, 6.7)	2002–2020	−2.3 (−2.7, −1.9)^a^	NA	NA	NA	NA	−1.9 (−2.6, −1.1)^a^	Reference
NH Black or African American	1999–2015	−2.9 (−3.3, −2.5)^a^	2015–2020	4.4 (1.8, 7.1)^a^	NA	NA	NA	NA	−1.2 (−1.8, −0.6)^a^	0.001
NH American Indian or Alaska Native	1999–2020	0.3 (−0.5, 1.2)	NA	NA	NA	NA	NA	NA	0.3 (−0.5, 1.2)	0.056
NH Asian or Pacific Islander	1999–2020	−0.03 (−1.1, 1.1)	NA	NA	NA	NA	NA	NA	−0.03 (−1.1, 1.1)	0.022
Hispanic or Latino	1999–2005	0.2 (−2.5, 3.0)	2005–2009	−7.7 (−14.5, −0.3)^a^	2009–2018	0.8 (−0.9, 2.5)	2018–2020	18.7 (4.0, 35.3)^a^	0.5 (−1.5, 2.5)	<0.001
Region										
Large metropolitan	1999–2003	−0.8 (−2.8, 1.3)	2003–2010	−5.5 (−6.7, −4.3)^a^	2010–2018	−1.4 (−2.5, −0.2)^a^	2018–2020	8.5 (−0.3, 18.1)	−1.8 (−2.7, −0.8)^a^	Reference
Medium/small metropolitan	1999–2003	−0.003 (−2.4, 2.4)	2003–2018	−2.6 (−3.0, −2.2)^a^	2018–2020	10.3 (0.7, 20.9)^a^	NA	NA	−1.0 (−1.9, −0.02)^a^	<0.001
Nonmetropolitan	1999–2020	−1.2 (−1.5, −0.8)^a^	NA	NA	NA	NA	NA	NA	−1.2 (−1.5, −0.8)^a^	<0.001

Segments (periods 1–4) were identified by Joinpoint regressions. Joinpoint regression was conducted for each stratum and therefore there could be different numbers of segments (from 1–4) for each stratum.

*APC* annual percentage change, *AAPC* average annual percent change, *NA* not applicable, *NH* non-Hispanic.

^a^Indicates that the APC or AAPC is significantly different from 0 at α = 0.05.

### Sex-stratified analysis

When stratified by sex, men had consistently higher AAMRs than women (overall AAMR men: 4.49; 95% CI: 4.45–4.52; women: 1.84; 95% CI: 1.81–1.86). There was a general decrease in AAMR from 1999 to 2018 followed by an increase from 2018 to 2020 (Fig. 1). The AAMR of young men remained stable from 1999 to 2003 (APC: 0.3%; 95% CI: -1.5% to 2.1%); steeply decreased from 2003 to 2009 (APC: -4.4%; 95% CI: -5.6% to -3.1%); decreased from 2009 to 2018 (APC: -2.2%; 95% CI: -2.9% to -1.5%); and then increased from 2018 to 2020 (APC: 7.5%; 95% CI: 0.6% to 14.9%). The AAMR of young women decreased from 1999 to 2018 (APC: -2.1%; 95% CI: -2.5% to -1.7%) and notably increased from 2018 to 2020 (APC: 9.6%; 95% CI: -5.9% to 27.7%). The AAPC in age-adjusted AMI mortality rate was -1.5% per year (95% CI: -2.2% to -0.7%) for young men and -1.0% per year (95% CI: -2.4% to -0.4%), respectively (Table 1).

### Race/ethnicity stratified analysis

**Fig. 2:**
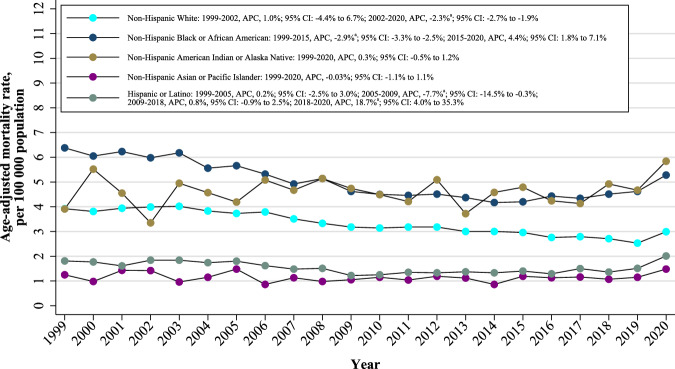
Race/ethnicity-stratified trends of age-adjusted acute myocardial infarction-related mortality rates among adults aged 15–44 years from 1999 to 2020 in the United States. ^a^Indicates that the annual percentage change (APC) is significantly different from 0 at α = 0.05.

When stratified by race/ethnicity, AAMRs were highest among non-Hispanic Black or African American (overall AAMR: 5.07, 95% CI: 4.99–5.15) followed by non-Hispanic American Indian or Alaska Native (overall AAMR: 4.61, 95% CI: 4.32–4.90), non-Hispanic White (overall AAMR: 3.36, 95% CI: 3.34–3.39), Hispanic or Latino (overall AAMR: 1.51, 95% CI: 1.47–1.55), and non-Hispanic Asian or Pacific Islander (overall AAMR: 1.15, 95% CI: 1.10–1.21). The AAMRs for all racial/ethnic groups for AMI in young adults decreased from 1999 to 2018 and increased from 2018 to 2020 (Fig. 2). The AAMR for the non-Hispanic White population showed a slight increase from 1999 to 2002 (APC: 1.0%; 95% CI: -4.4% to 6.7%) and a decrease from 2002 to 2020 (APC: -2.3%; 95% CI: -2.7% to -1.9%), with AAPC of -1.9% (95% CI: -2.6% to -1.1%) (Fig. 2 and Table 1). The AAMR for non-Hispanic Black or African Americans decreased from 1999 to 2015 (APC: -2.9%; 95% CI: -3.3% to -2.5%), followed by an increase until 2020 (APC: 4.4%; 95% CI: 1.8% to 7.1%), with AAPC of -1.2% (95% CI: -1.8% to -0.6%). The AAMRs for non-Hispanic American Indian or Alaska Native (APC: 0.3%; 95% CI: -0.5% to 1.2%) and non-Hispanic Asian or Pacific Islander (APC: -0.03%; 95% CI: -1.1% to 1.1%) were stable, respectively. The AAMR for the Hispanic or Latino population was stable from 1999 to 2005 (APC: 0.2%; 95% CI: -2.5% to 3.0%); decreased from 2005 to 2009 (APC: -7.7%; 95% CI: -14.5% to -0.3%); slightly increased from 2009 to 2018 (APC: 0.8%, 95% CI: -0.9% to 2.5%); and sharply increased from 2018 to 2020 (APC: 18.7%; 95% CI: 4.0% to 35.3%), with AAPC of 0.5% (95% CI: -1.5% to 2.5%).

### Geographic region analysis

**Fig. 3:**
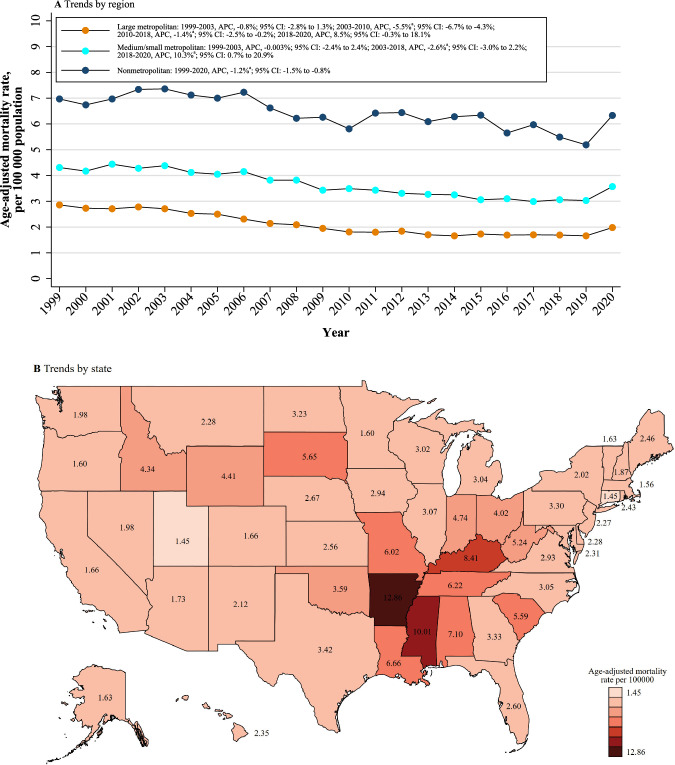
Geographic stratified analysis of acute myocardial infarction-related age-adjusted mortality rates among adults aged 15–44 years from 1999 to 2020 in the United States. **A** Stratified by region. **B** Stratified by state. ^a^Indicates that the annual percentage change (APC) is significantly different from 0 at α = 0.05.

Nonmetropolitan areas had consistently higher AMI-related AAMRs than medium/small or large metropolitan areas throughout the study period, with overall AAMRs of 6.49 (95% CI: 6.41–6.58), 3.68 (95% CI: 3.64–3.73), and 2.13 (95% CI: 2.11–2.15), respectively. AAMRs of large and medium/small metropolitan areas declined from 1999 to 2018 and notably increased from 2018 to 2020. AAMRs of nonmetropolitan areas tended to decrease from 1999 to 2020 (APC: -1.2%; 95% CI: -1.5% to-0.8%). (Fig. 3A and Table 1).

There were significant differences in the burden of AMI-related mortality between states, with the AAMRs ranging from 1.45 (95% CI: 1.31–1.58) in Connecticut to 12.86 (95% CI: 12.40–13.33) in Arkansas (Fig. 3B). States that fell into the top 90th percentile were Louisiana, Alabama, Kentucky, Mississippi, and Arkansas, while states that fell into the lowest 10th percentile, namely, Connecticut, Utah, Massachusetts, Oregon, Minnesota, and Vermont (Supplementary Tables 5). On average, the highest mortality over the study period was observed in the Southern region (AAMR: 4.26; 95% CI: 4.22–4.30), followed by the Midwestern (AAMR: 3.51; 95% CI: 3.46–3.56), Northeastern (AAMR: 2.28: 95% CI: 2.23–2.32), and Western (AAMR: 1.84; 95% CI: 1.80–1.87) regions (Supplementary Table 6).

## Discussion

Our data demonstrate that from 1999 to 2020, there was a general decrease in AMI-related mortality rates in young adults while the decreasing trend was more obvious after 2003. The sex-stratified AAMR trends were generally consistent with the overall trend. There are notable differences by race/ethnicity, namely increasing AAMRs (especially in Non-Hispanic Black or African Americans) after 2015, which was not as apparent among the Non-Hispanic White population. AMI-related mortality also significantly differed by geographical region and degree of urbanization. Finally, we observed a relatively uniform increase in AMI-related mortality in the first year of the COVID-19 pandemic. This study adds to existing evidence by specifically highlighting demographic disparities in AMI mortality trends, including sex, race/ethnicity, and geographic region, which have not been comprehensively explored in young adults. These findings underscore the need for targeted public health strategies to address the unique risk factors and healthcare access barriers faced by this demographic. Moreover, the impact of the COVID-19 pandemic on AMI mortality rates emphasizes the vulnerability of young adults during systemic healthcare disruptions. By identifying these trends and disparities, our study provides a foundation for developing gender- and race-specific prevention and intervention programs and improving healthcare infrastructure to ensure equitable access to care for young adults.

The overall decreasing trend in AMI-related mortality in young adults is consistent with previous studies before 2020^5,18^. There was an overlap between the period (2003 to 2010) of a steeper decrease in overall AAMR trends and the period (2004–2012) with marked improvement in the quality of care from the launching of Centers for Medicare and Medicaid Services (CMS) Hospital Inpatient Quality Reporting program^19,20^. The pay-for-performance and the public reporting for the quality of care of acute myocardial infarction might be important factors for the considerable decrease in AMI-related mortality in young adults. Furthermore, the expansion of Medicaid coverage starting in 2014 due to the Patient Protection and Affordable Care Act also notably reduced the cardiovascular mortality of adults aged 25–64 years^21,22^. The decrease in cardiovascular mortality might come from the improvements in the screening and prevention of CVD from the Medicaid expansion^23^. There is, however, no obvious decrease in the hospitalization rate of AMI in young adults, even a slight increase in young female patients, compared with the overall decreasing trend for middle-aged and elderly patients^5,24,25^.

The less significant decrease in AMI-related mortality rates after 2010 might be due to the unchanged or even increased prevalence of atherosclerosis in young adults. The study by George et al.^26^ show that the hospitalization rates of acute ischemic stroke, another major adverse event of atherosclerosis, increased by 35.6% for US adults aged 35–44 years from 2003–2004 to 2011–2012. As major risk factors for atherosclerosis, diabetes, and obesity increased while hypertension did not change for US young adults aged 20–44 years from 2009–2020^27^. Although rupture of the atherosclerotic plaque is the predominant cause of AMI, there are other non-atherosclerotic or non-atherothrombotic causes of insufficient blood and/or oxygen supply to the myocardium leading to AMI. The majority of myocardial injuries from non-atherosclerotic or non-atherothrombotic causes (e.g., tachyarrhythmia, coronary artery spasm, coronary dissection, hypoxemia, anemia, etc.) are classified as type 2 myocardial infarction from the Fourth Universal Definition of Myocardial Infarction^28^. DeFilippis et al. have shown that the prevalence of type 2 myocardial infarction can vary from 2%–58% in patients with myocardial infarction while the proportion changes to 26%–58% for patients with myocardial infarction and presented in the emergency department^29^. In contrast to the atherothrombotic cause (type 1 myocardial infarction), the causes of type 2 myocardial infarction are a collection of mechanisms. Some mechanisms of type 2 myocardial infarction are neither preventable nor socio-economically beneficial compared with atherosclerosis, the only mechanism of type 1 myocardial infarction^30^. As a result, this fact may also account for the deceleration of the decrease of AMI-related mortality as a proportion of the AMI-related mortality is currently less preventable.

The more considerable decrease in AMI-related mortality among Non-Hispanic Blacks than other races or ethnicities before 2015 may be partly related to the CMS Hospital Inpatient Quality Reporting program. A study from Trivedi et al.^31^ has shown an improvement in the treatment of AMI between White and Black patients with declines in both between-and within-hospital differences after launching the program. Although similar improvement has been shown between White and Hispanic patients, results from this study show a less obvious decrease in AMI-related mortality in the Hispanic or Latino population. Besides, the AMI-related hospitalization rate of the young Black population is generally less than that of the young White population^5,24^. Further study may be required to investigate the phenomenon that higher AMI-related mortality of young Non-Hispanic Black or African Americans but with potentially less AMI-related hospitalization rate compared with the Non-Hispanic White population. Moreover, the expansion of Medicaid coverage may have less effect on Non-Hispanic Blacks than the CMS Hospital Inpatient Quality Reporting program. Although Lee et al. mention reductions in all-cause mortality of Non-Hispanic Blacks due to the expansion of Medicaid coverage from 2014–2018^21^, our study observes an increase in AAMR of Non-Hispanic Blacks from 2015–2020. This may come from the inconsistent improvement of utilization of the acute and critical care procedures for Medicaid-covered patients and not all states participating in the Affordable Care Act^23,32^. Socioeconomic factors, such as inequalities in income, education, and employment, may also contribute to the observed disparities. As reported by Lopez‑Neyman et al. in a study of 8,370 US adults aged ≥20 years from the National Health and Nutrition Examination Survey (NHANES) 2011–2018, Non-Hispanic Black and Hispanic Americans were the leading groups for cardiovascular disease (CVD) risk factors, such as obesity and diabetes. These groups also had the lowest proportion of individuals with education levels ≥Bachelor’s degree and the highest proportion living with a low poverty income ratio (<1.3)^33^. Furthermore, structural inequities resulting from residential segregation may also explain these differences. For example, Black and Hispanic individuals are more likely to reside in poverty-stricken areas. These neighborhoods often experience limited infrastructure investment and a poorer physical and mental health environment, both of which increase the risk of CVD^34^.

The geographical variations of AMI-related mortality in young adults are considerable with high AAMRs concentrated in the Southern region. This observation is consistent with the previous study of stroke-related mortality in young and middle-aged adults (aged 25–64 years)^35^ and the prevalence of pre-existing CVD burden in this region^36,37^. Apart from the state-wise comparison, the differences in AMI-related mortality in young adults among different city sizes indicate the possible county-wise disparities in medical resources^38^. A study by Miller et al. further highlights the urgency of addressing healthcare disparities in the Southern rural region, which is worse than both the national rural average and southern urban areas^39^. For instance, in Southern rural areas, the uninsured population under 65 years of age is 79% higher compared to the healthiest rural regions in the United States. Additionally, the number of physicians per one million population drops from 370 in Southern urban areas to just 68 in Southern rural areas, a decline of 82%.

The outbreak of the COVID-19 pandemic has substantially challenged the healthcare system globally. There was a significant reduction in hospital admissions for non-COVID-19 illnesses during the initial phase of the pandemic^40^. Lockdown measures designed to contain the scope of COVID-19 infection may have also dissuaded or delayed seeking out care in patients who were experiencing AMI^41^. The surge of AMI deaths at the home of young patients may show a clue of the overwhelming of the healthcare system and potential age differentiation during the pandemic. Infection with SARS-CoV-2 has also been associated with an increased risk of AMI^42^. Moreover, there was a noticeable variation in mortality of different races/ethnicities. The surge in Non-Hispanic Black or African American and Hispanic or Latino populations was higher than the Non-Hispanic White population, which has been previously observed.

The sex difference in overall AMI-related mortality rates is also reported by Millett et al., who examined MI incidence among 471,998 participants from the UK Biobank^43^. They found that women had lower rates of MI, reporting a three-fold higher MI incidence in men compared to women. However, there is no evidence that CVD risk factors are more strongly correlated with MI in men. On the contrary, several risk factors were more strongly associated with MI in women, leading them to predict a potential catch-up in MI incidence among women. Moreover, Millett et al. emphasized the importance of equitable access to treatment and support for lowering CVD risk factors among women. Similarly, a study by Lichtman et al., involving nearly 3,000 patients aged 18–55 years hospitalized for AMI in the US, showed no sex differences in the presentation of AMI symptoms^44^. However, both women and their healthcare providers were less likely to attribute prodromal symptoms to heart disease compared to men, potentially contributing to delayed diagnosis and treatment in women.

Our study has several limitations. Use of death certificate information may lead to misclassification of the true cause(s) of death as well as incompleteness or inaccuracies in the reporting of certain demographic information, such as race/ethnicity^35^. De Henauw et al.^45^ reported the misclassification (about 27.1% and 38.2% from two centers) of the AMI to coronary heart disease in death certificates in the WHO-MONICA Ghent-Charleroi Study. While Lloyd-Jones et al.^46^ found the overrepresentation (about 24.2%) of coronary heart disease on death certificates in the Framingham Heart Study. Moreover, there is a lack of information about the underlying etiology, presentations, subtypes and treatments of the AMI that preceded the deaths in the present study. Further information can help to improve the risk assessment and treatment strategy for AMI among young adults. The geographical differences might be affected by the migration of patients among states.

In conclusion, while there was a general decrease in the mortality rates of young adults from 1999 to 2020, the slowing decline and the slight rise during the first year of the COVID-19 pandemic underscore the ongoing challenges in addressing AMI-related mortality in this demographic. This study highlights significant disparities by sex, racial/ethnic group, geographic location, and rural/urban status, emphasizing the critical need for targeted and equitable public health interventions. Future research should focus on addressing these disparities, improving healthcare access, and tailoring prevention strategies to meet the specific needs of young adults, ultimately reducing AMI-related mortality and promoting health equity.

## Supplementary information

Supplement 0103

## Data Availability

The dataset is publicly available on the CDC WONDER webpage (https://wonder.cdc.gov/).

## Code availability

Codes for statistical analysis of this study are available upon request from Haibin Li.
