# Supplementary material for: Demographic and regional trends of acute myocardial infarction-related mortality among young adults in the US, 1999–2020
Source: NPJ Cardiovasc Health. 2025 Mar 4;2:9. doi: 10.1038/s44325-025-00046-w (PMC12912338; doi:10.1038/s44325-025-00046-w)
Supplement: Supplementary file 1 — Supplement 0103 [file 44325_2025_46_MOESM1_ESM.pdf]

**Supplementary Table 1**. **Absolute Number of Acute Myocardial Infarction Related Deaths Stratified by Sex and Race and Ethnicity in the United States, 1999-2020.**									
	**Overall**	**Women**	**Men**	**NH White**	**NH Black or African American**	**NH American Indian or Alaska Native**	**NH Asian or Pacific Islander**	**Hispanic**	**Population**
**Year**	**Deaths**	**Deaths**	**Deaths**	**Deaths**	**Deaths**	**Deaths**	**Deaths**	**Deaths**	**Total**
1999	4732	1317	3415	3379	968	39	67	261	123931114
2000	4566	1233	3333	3250	925	56	54	265	124224142
2001	4674	1298	3376	3312	960	46	80	259	124736844
2002	4658	1328	3330	3313	916	34	81	301	124844430
2003	4614	1299	3315	3243	938	49	59	312	124786855
2004	4342	1215	3127	3056	851	45	71	309	125014943
2005	4222	1167	3055	2901	861	41	93	324	125210354
2006	4139	1161	2978	2905	802	49	57	315	125482824
2007	3802	1095	2707	2633	744	45	79	294	125655508
2008	3635	1054	2581	2431	780	49	69	300	125791451
2009	3376	983	2393	2297	693	45	76	258	125788085
2010	3252	943	2309	2184	667	41	84	270	125760896
2011	3254	919	2335	2183	660	39	77	290	126216327
2012	3287	972	2315	2180	671	48	91	289	126769646
2013	3108	902	2206	2011	661	34	90	303	127251679
2014	3056	933	2123	1996	631	42	71	305	128009458
2015	3107	969	2138	1978	642	44	101	330	128575201
2016	2950	880	2070	1826	678	40	95	307	128658426
2017	3000	913	2087	1812	678	40	99	365	129468337
2018	2999	908	2091	1801	709	49	98	340	129946462
2019	2947	894	2053	1690	732	47	105	365	130286975
2020	3552	1091	2461	1998	855	58	138	497	130761522
**Total**	81272	23474	57798	54379	17022	980	1835	6859	2787171479

NH: Non-Hispanic

**Supplementary Table 2. Trend in Acute Myocardial Infarction Related Deaths Stratified by Place of Death in the United States, 1999-2020.**				
	**Medical Facility**	**Nursing Home/Long-term Care Facility**	**Hospices**	**Home**
**Year**	**Deaths**	**Deaths**	**Deaths**	**Deaths**
1999	3378	80	NA	1051
2000	3263	72	NA	1012
2001	3286	82	NA	1093
2002	3182	86	NA	1127
2003	3129	76	NA	1158
2004	2897	69	NA	1115
2005	2802	72	NA	1082
2006	2704	69	NA	1114
2007	2467	61	15	1061
2008	2340	57	11	977
2009	2110	45	12	907
2010	2120	36	12	857
2011	2102	49	18	897
2012	2165	43	16	883
2013	1983	39	16	905
2014	1896	36	19	900
2015	1954	40	12	895
2016	1827	43	19	875
2017	1877	34	19	891
2018	1835	51	24	890
2019	1792	42	19	881
2020	2111	33	24	1138
**Total**	53220	1215	248	21709

**Supplementary Table 3. Acute Myocardial Infarction Related Mortality for age group 25-44 years in the United States, 1999-2020**				
Year	Death	Population	Crude Rate	Age Adjusted Rate
1999	4,631	85,255,083	5.43	5.54
2000	4,458	85,040,251	5.24	5.38
2001	4,555	84,523,274	5.39	5.47
2002	4,534	83,990,295	5.4	5.48
2003	4,490	83,398,001	5.38	5.48
2004	4,223	83,066,831	5.08	5.2
2005	4,090	82,764,185	4.94	5.05
2006	4,033	82,638,980	4.88	4.99
2007	3,705	82,509,693	4.49	4.62
2008	3,521	82,399,959	4.27	4.45
2009	3,278	82,211,153	3.99	4.19
2010	3,148	82,134,554	3.83	4.04
2011	3,155	82,418,452	3.83	4.14
2012	3,176	82,825,741	3.83	4.13
2013	3,012	83,297,277	3.62	3.92
2014	2,968	84,029,637	3.53	3.83
2015	3,005	84,726,985	3.55	3.87
2016	2,872	85,147,399	3.37	3.72
2017	2,897	86,218,042	3.36	3.72
2018	2,921	86,975,662	3.36	3.71
2019	2,867	87,599,465	3.27	3.61
2020	3,464	88,205,838	3.93	4.29
Total	79,003	1,851,376,757	4.27	4.52

**Supplementary Table 4. Acute Myocardial Infarction Mortality (listed as underlying cause), among young adults in the United States, 1999-2020**				
Year	Death	Population	Crude Rate	Age Adjusted Rate
1999	3743	123931114	3.02	3.09
2000	3559	124224142	2.86	2.88
2001	3715	124736844	2.98	3.04
2002	3623	124844430	2.90	3.02
2003	3580	124786855	2.87	3.01
2004	3336	125014943	2.67	2.79
2005	3194	125210354	2.55	2.72
2006	3158	125482824	2.52	2.68
2007	2856	125655508	2.27	2.43
2008	2723	125791451	2.16	2.32
2009	2489	125788085	1.98	2.17
2010	2467	125760896	1.96	2.17
2011	2482	126216327	1.97	2.17
2012	2459	126769646	1.94	2.14
2013	2356	127251679	1.85	2.06
2014	2264	128009458	1.77	1.99
2015	2292	128575201	1.78	1.99
2016	2154	128658426	1.67	1.89
2017	2180	129468337	1.68	1.92
2018	2149	129946462	1.65	1.84
2019	2033	130286975	1.56	1.74
2020	2404	130761522	1.84	2.06
Total	61216	2787171479	2.20	2.36

**Supplementary Table 5. Acute Myocardial Infarction Related Mortality Stratified by States in the United States, 1999-2020**						
State	Rank	Percentile	Age Adjusted Mortality Rate per 100,000	S.E.	Z value	*P* value
Connecticut (09)	1	1	1.45	0.24	-45.10	<0.001
Utah (49)	2	2	1.45	0.24	-45.64	<0.001
Massachusetts (25)	3	4	1.56	0.24	-46.09	<0.001
Oregon (41)	4	6	1.60	0.24	-45.52	<0.001
Minnesota (27)	5	8	1.60	0.24	-45.04	<0.001
Vermont (50)	6	10	1.63	0.24	-37.43	<0.001
Alaska (02)	7	12	1.63	0.24	-38.93	<0.001
California (06)	8	14	1.66	0.24	-45.27	<0.001
Colorado (08)	9	16	1.66	0.24	-46.51	<0.001
Arizona (04)	10	18	1.73	0.24	-44.99	<0.001
New Hampshire (33)	11	20	1.87	0.24	-40.26	<0.001
Washington (53)	12	22	1.98	0.24	-42.45	<0.001
Nevada (32)	13	24	1.98	0.24	-43.98	<0.001
District of Columbia (11)	14	26	2.02	0.24	-44.55	<0.001
New York (36)	15	28	2.02	0.24	-35.41	<0.001
New Mexico (35)	16	30	2.12	0.24	-40.03	<0.001
New Jersey (34)	17	32	2.27	0.24	-43.20	<0.001
Delaware (10)	18	34	2.28	0.24	-35.97	<0.001
Montana (30)	19	36	2.28	0.24	-35.27	<0.001
Maryland (24)	20	38	2.31	0.24	-42.20	<0.001
Hawaii (15)	21	40	2.35	0.24	-37.14	<0.001
Rhode Island (44)	22	42	2.43	0.24	-35.46	<0.001
Maine (23)	23	44	2.46	0.24	-36.75	<0.001
Kansas (20)	24	46	2.56	0.24	-39.01	<0.001
Florida (12)	25	48	2.60	0.24	-42.17	<0.001
Nebraska (31)	26	50	2.67	0.24	-36.67	<0.001
Virginia (51)	27	51	2.93	0.24	-39.72	<0.001
Iowa (19)	28	53	2.94	0.24	-37.57	<0.001
Wisconsin (55)	29	55	3.02	0.24	-38.90	<0.001
Michigan (26)	30	57	3.04	0.24	-39.69	<0.001
North Carolina (37)	31	59	3.05	0.24	-39.65	<0.001
Illinois (17)	32	61	3.07	0.24	-39.93	<0.001
North Dakota (38)	33	63	3.23	0.24	-27.79	<0.001
Pennsylvania (42)	34	65	3.30	0.24	-38.64	<0.001
Georgia (13)	35	67	3.33	0.24	-38.52	<0.001
Texas (48)	36	69	3.42	0.24	-38.80	<0.001

Oklahoma (40)	37	71	3.59	0.24	-35.11	<0.001
Ohio (39)	38	73	4.02	0.24	-35.36	<0.001
Idaho (16)	39	75	4.34	0.24	-27.83	<0.001
Wyoming (56)	40	77	4.41	0.24	-21.12	<0.001
Indiana (18)	41	79	4.74	0.24	-31.68	<0.001
West Virginia (54)	42	81	5.24	0.24	-24.89	<0.001
South Carolina (45)	43	83	5.59	0.24	-27.09	<0.001
South Dakota (46)	44	85	5.65	0.24	-18.77	<0.001
Missouri (29)	45	87	6.02	0.24	-25.91	<0.001
Tennessee (47)	46	89	6.22	0.24	-25.15	<0.001
Louisiana (22)	47	91	6.66	0.24	-22.72	<0.001
Alabama (01)	48	93	7.10	0.24	-20.73	<0.001
Kentucky (21)	49	95	8.41	0.24	-15.72	<0.001
Mississippi (28)	50	97	10.01	0.24	-8.94	<0.001
Arkansas (05)	51	99	12.86	0.24	Reference	Reference

**Supplementary Table 6. Trend in Acute Myocardial Infarction Related Deaths Stratified by Region in the United States, 1999-2020.**					
**Census Region**	**Year**	**Deaths**	**Population**	**Crude Rate**	**Age Adjusted Rate**
Northeast	1999	683	23195712	2.94	2.87
Northeast	2000	701	23129640	3.03	2.97
Northeast	2001	710	23076376	3.08	3.02
Northeast	2002	680	22999073	2.96	2.9
Northeast	2003	653	22910174	2.85	2.83
Northeast	2004	596	22771204	2.62	2.67
Northeast	2005	535	22631947	2.36	2.43
Northeast	2006	536	22503173	2.38	2.43
Northeast	2007	518	22386082	2.31	2.43
Northeast	2008	497	22310165	2.23	2.35
Northeast	2009	441	22219222	1.98	2.14
Northeast	2010	379	22143914	1.71	1.84
Northeast	2011	386	22078907	1.75	1.91
Northeast	2012	416	22095492	1.88	2.09
Northeast	2013	386	22071211	1.75	1.96
Northeast	2014	338	22101076	1.53	1.71
Northeast	2015	355	22066680	1.61	1.81
Northeast	2016	326	21952203	1.49	1.71
Northeast	2017	304	21994516	1.38	1.55
Northeast	2018	289	21782384	1.33	1.48
Northeast	2019	309	21707127	1.42	1.63
Northeast	2020	367	21635983	1.7	1.91
Northeast	Total	10405	491762261	2.12	2.28
Midwest	1999	1228	28157540	4.36	4.39
Midwest	2000	1182	28106978	4.21	4.26
Midwest	2001	1220	27999506	4.36	4.49
Midwest	2002	1100	27838875	3.95	4.06
Midwest	2003	1083	27677551	3.91	4.1
Midwest	2004	1050	27528941	3.81	4.01
Midwest	2005	986	27369288	3.6	3.84
Midwest	2006	983	27218729	3.61	3.89
Midwest	2007	890	27052214	3.29	3.53
Midwest	2008	842	26873600	3.13	3.43
Midwest	2009	799	26686256	2.99	3.3
Midwest	2010	782	26560588	2.94	3.28
Midwest	2011	774	26511660	2.92	3.3
Midwest	2012	727	26478021	2.75	3.09
Midwest	2013	714	26510344	2.69	3.07

Midwest	2014	686	26526418	2.59	2.97
Midwest	2015	730	26508037	2.75	3.1
Midwest	2016	652	26437986	2.47	2.86
Midwest	2017	681	26474523	2.57	2.94
Midwest	2018	692	26540529	2.61	2.93
Midwest	2019	634	26560540	2.39	2.67
Midwest	2020	747	26568716	2.81	3.1
Midwest	Total	19182	594186840	3.23	3.51
South	1999	2234	44143240	5.06	5.18
South	2000	2105	44364783	4.74	4.89
South	2001	2172	44687448	4.86	5.02
South	2002	2292	44860910	5.11	5.32
South	2003	2275	44960227	5.06	5.28
South	2004	2123	45298675	4.69	4.91
South	2005	2145	45620044	4.7	4.99
South	2006	2137	45951490	4.65	4.95
South	2007	1887	46262324	4.08	4.36
South	2008	1814	46481013	3.9	4.25
South	2009	1663	46641383	3.57	3.89
South	2010	1634	46743966	3.5	3.82
South	2011	1621	47061985	3.44	3.82
South	2012	1681	47424120	3.54	3.93
South	2013	1555	47710313	3.26	3.63
South	2014	1639	48133644	3.41	3.79
South	2015	1572	48510323	3.24	3.6
South	2016	1527	48668525	3.14	3.53
South	2017	1585	49080042	3.23	3.6
South	2018	1574	49448163	3.18	3.53
South	2019	1550	49687076	3.12	3.47
South	2020	1866	50085949	3.73	4.1
South	Total	40651	1031825643	3.94	4.26
West	1999	587	28434622	2.06	2.14
West	2000	578	28622741	2.02	2.07
West	2001	572	28973514	1.97	2.09
West	2002	586	29145572	2.01	2.14
West	2003	603	29238903	2.06	2.21
West	2004	573	29416123	1.95	2.09
West	2005	556	29589075	1.88	2.02
West	2006	483	29809432	1.62	1.77
West	2007	507	29954888	1.69	1.88
West	2008	482	30126673	1.6	1.76

West	2009	473	30241224	1.56	1.69
West	2010	457	30312428	1.51	1.7
West	2011	473	30563775	1.55	1.73
West	2012	463	30772013	1.5	1.69
West	2013	453	30959811	1.46	1.66
West	2014	393	31248320	1.26	1.4
West	2015	450	31490161	1.43	1.58
West	2016	445	31599712	1.41	1.58
West	2017	430	31919256	1.35	1.52
West	2018	444	32175386	1.38	1.51
West	2019	454	32332232	1.4	1.51
West	2020	572	32470874	1.76	1.92
West	Total	11034	669396735	1.65	1.84
Total		81272	2787171479	2.92	3.15

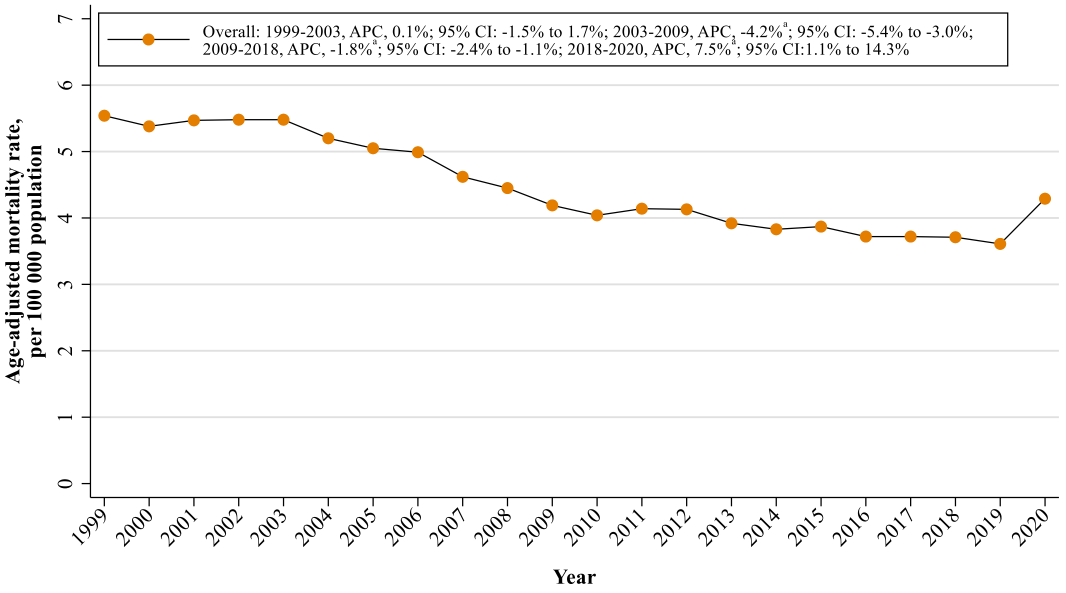

Supplementary Figure 1. Trends in acute myocardial infarction related age adjusted mortality rates for age group 25-44 years in the United States, 1999-2020.

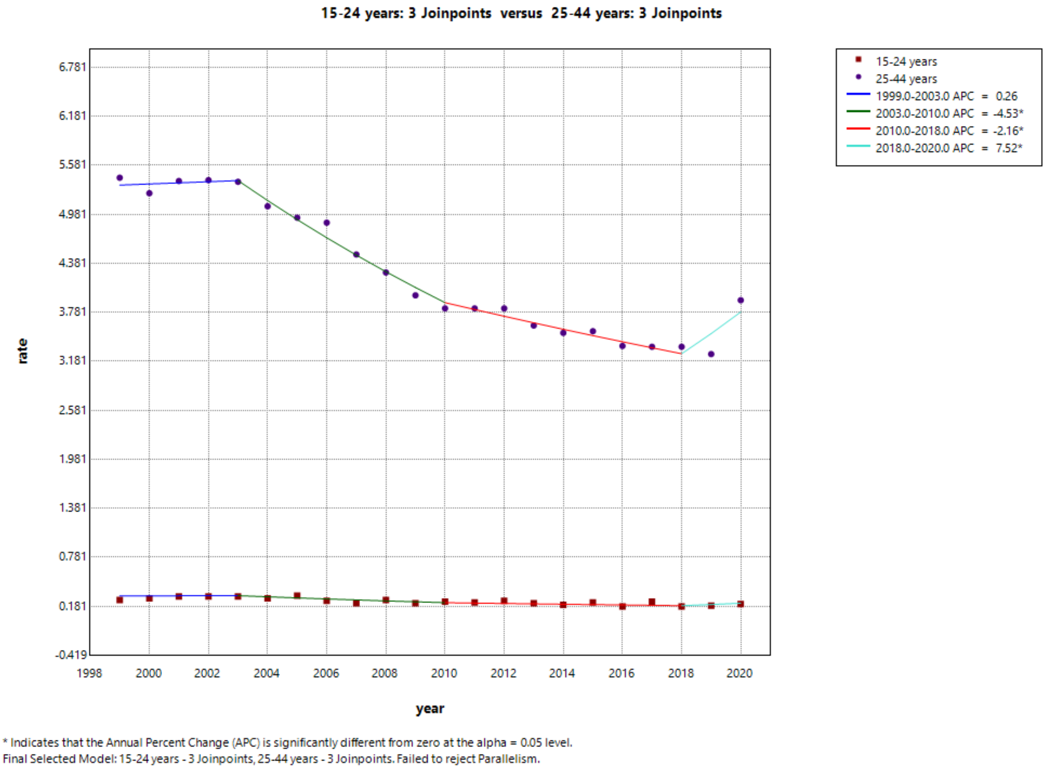

Graph **Data** **Model Estimates** **Trends** **Model Selection** **Pairwise Comparison**

Test For Parallelism

Kmax^	Numerator Degrees of Freedom	Denominator Degrees of Freedom	Number of Permutations	P-Value	Significance Level~
3 Joinpoint(s)	7	28	4500	0.455556	0.0500000

Final Selected Model: Failed to reject Parallelism
~ Significance level for individual test
^ Assumed number of joinpoints for the comparison (parallel or coincident) test Learn More

Supplementary Figure 2. Trends and pairwise comparison in acute myocardial infarction related crude mortality rates for age group 15-24 years and 25-44 years in the United States, 1999-2020

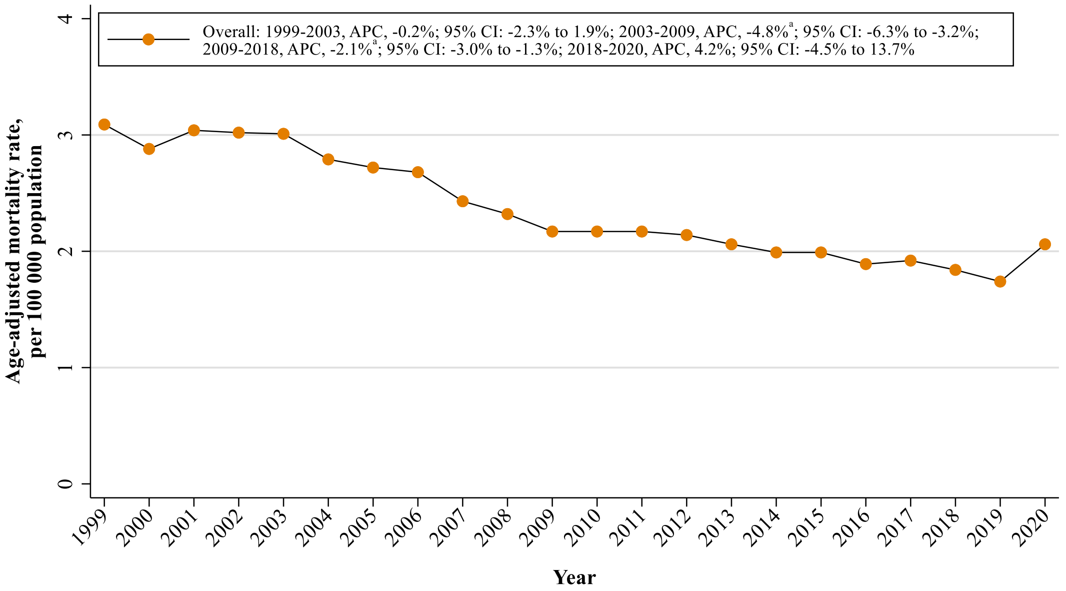

Supplementary Figure 3. Trends in acute myocardial infarction (listed as underlying cause), among young adults in the United States, 1999-2019
